# Child Death and Mothers’ Subsequent Mental Health in a High-Mortality African Community

**DOI:** 10.1111/padr.12682

**Published:** 2024-11-24

**Authors:** Emily Smith-Greenaway, Abigail Weitzman, Eric Lungu

**Affiliations:** Department of Sociology, Dana and David Dornsife College of Letters Arts and Sciences, University of Southern California, Los Angeles, CA 90089, USA.; Department of Sociology, College of Liberal Arts, The University of Texas at Austin, Austin, TX 78712, USA.; Partners in Hope, Lilongwe, Malawi.

## Abstract

Despite declines in child mortality rates, experiencing a child death remains a common feature of motherhood in many contemporary African populations. Yet, we lack population insights into the consequences of child death for mothers’ well-being in the high-mortality regions where it concentrates. Contrasting an extensive psychology literature on the severe and long-lasting consequences of child death for parents in low-mortality settings, a long-standing thesis in multiple social science literature is that the normativity of child death in high-mortality settings can lead to a numbing effect—muting parents’ reactions to child loss. Yet, select anthropological accounts challenge this thesis, arguing instead that child death can also bear notable consequences for bereaved parents in communities where it is common. This study brings population data to bear, analyzing two representative samples of women in Balaka, Malawi, to examine if child death has measurable mental health consequences for mothers, including elevated and/or worsening depressive symptoms. Further, the study explores the potential influence of children’s near-death experiences on mothers. The results offer evidence that child loss—and the ever-present threat of it—are underappreciated drivers of women’s poor mental health, and overall well-being.

## Introduction

The death of a child remains a common parental experience for contemporary cohorts of mothers in sub-Saharan Africa (Hailu, Ketema, and Beyene 2021; Smith-Greenaway et al. 2021; Smith-Greenaway and Sennott 2016; Smith-Greenaway and Trinitapoli 2020; Weitzman and Smith-Greenaway 2020). Despite dramatic declines in the risk of child death over the past 50 years (You et al. 2015), the combination of persistently high fertility and mortality mean that in many contemporary African populations, upward of one-third to one-half of mothers (age 45–49 years old) have experienced one or more of their children die during early childhood (Smith-Greenaway et al. 2021).

Social science literature is replete with competing perspectives on what this high rate of child loss means for parents. A dominant perspective among social historians and anthropologists is that in high-mortality settings, child death is a routine, even anticipated, event that is of little consequence to parents. Coined the “neglect thesis” (Einarsdóttir 2005), this perspective contends that the normativity of child death in high-mortality populations leads parents to preemptively detach from their children and not intensely mourn their deaths. Child death is “viewed less as a tragedy than as a predictable and relatively minor misfortune, one to be accepted with equanimity and resignation as an unalterable fact of human existence” (Scheper-Hughes 1993, 275)—culminating in the bold claim that mothers experience “death without weeping” (Scheper-Hughes 1993). Yet, other anthropologists challenge this interpretation, emphasizing that child death is a devastating, disruptive experience for parents in high-mortality settings (see, e.g., Einarsdóttir 2005; M. Nations et al. 2015; Smørholm 2016). This latter perspective aligns with troves of studies by psychologists on bereaved parents in low-mortality contexts in contemporary North America and Western Europe, which document the significant effects of child death for bereaved parents—cataloging the severe and persistent consequences that bereaved parents, particularly mothers, endure (M. Stroebe, Schut, and Stroebe 2007; M. S. Stroebe et al. 2008; W. Stroebe and Schut 2001).

Despite this debate and the continued pervasiveness of child loss in African contexts, we lack comparable, population-based studies analyzing the consequences of child loss for parents’ mental well-being in these contexts. This is perhaps not surprising, given that demographers have only recently begun studying markers of mental health as a dimension of population health, especially in sub-Saharan Africa (Kohler et al. 2017). Instead, demographic research on the parental consequences of child mortality in high-mortality regions has focused almost exclusively on questions of how child death affects couples’ subsequent fertility (Bongaarts, Frank, and Lesthaeghe 1984; Defo 1998; Lindstrom and Kiros 2007; Preston 1978). This study broadens demographic scholarship by moving beyond the long-standing focus on the implications of child death for parents’ fertility to examine its consequences for parents’ mental health in contemporary Malawi.

**FIGURE 1:**
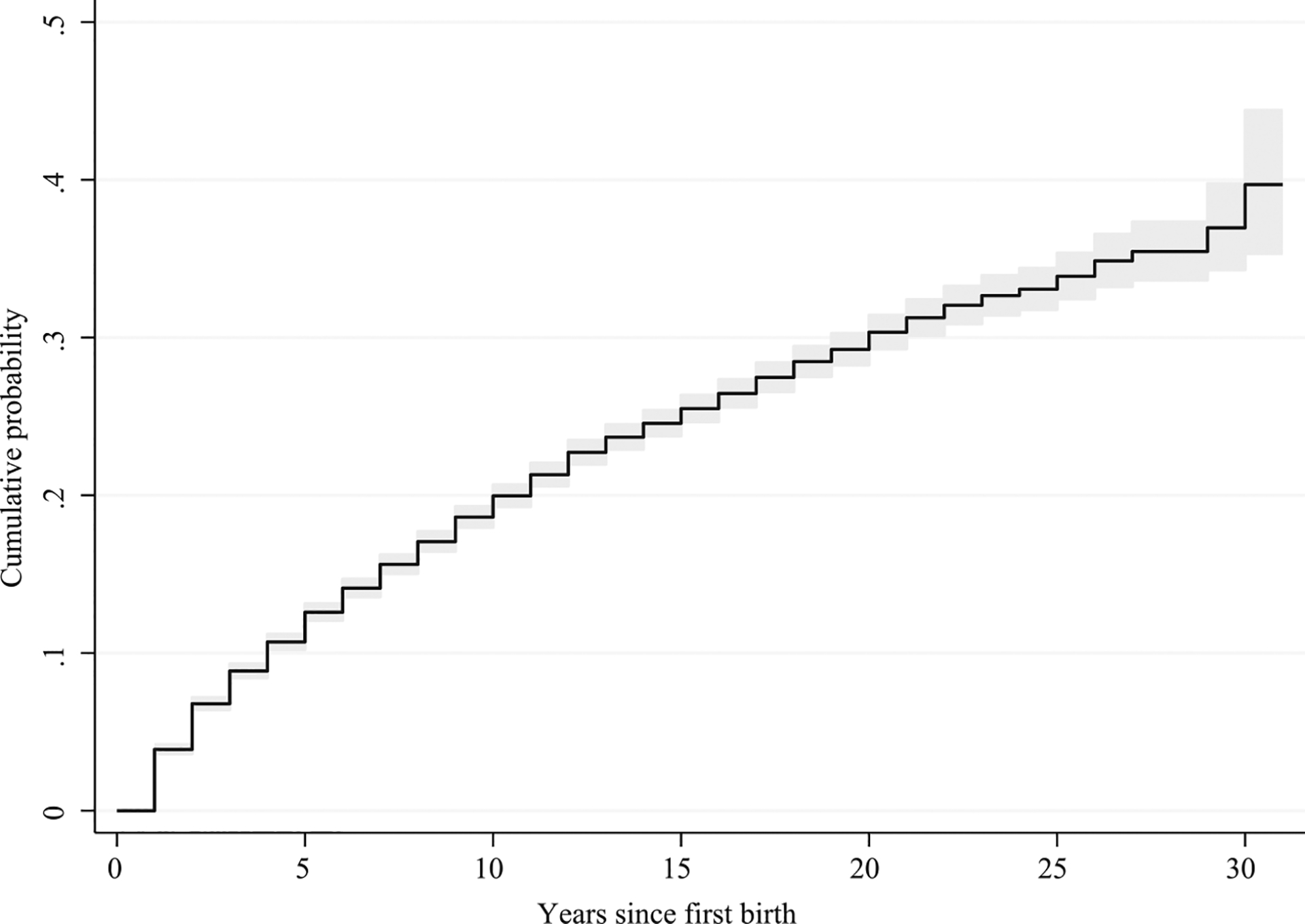
Cumulative probability of experiencing a child’s death among mothers in Malawi NOTE: Based on 2015 Malawi Demographic and Health Surveys data on women aged 15–49 with at least one child (*N* = 16,591). Cumulative probability of child death estimated from the time of mothers’ first birth. The shaded area represents a 95 percent confidence interval.

Child loss remains a prevalent feature of parenthood in Malawi. Figure 1 depicts the cumulative probability of experiencing a child’s death among reproductive-age mothers (age 15–49 years old) as a function of years since their first birth. According to Demographic and Health Survey (DHS) Program data, as recently as 2015, mothers had a 40 percent chance of experiencing a child’s death; this demonstrates the persistently high lifetime burden of child loss in this setting, and the need to understand its influence on parents’ well-being. Thus, we study the consequences of child death for mothers’ depressive symptoms. This dimension of women’s well-being remains minimally researched in this setting (Kohler et al. 2017), yet it is recognized—locally and globally—to be a major health threat (Jassi 2024; Prince et al. 2007). We further extend the literature beyond comparing only bereaved and non-bereaved mothers. Many women in this context have endured severe child health scares, as most child deaths stem from preventable causes induced by routine childhood illnesses. We thus explore if mothers’ perceived brushes with child death precipitate similar adversity, further testing the potential of psychological numbing.

### Perspectives of mothers’ experience of child death in high-mortality contexts

For much of human history, the death of a child was an ever-present part of life. As such, historians, anthropologists, and archaeologists have studied the experience extensively (Cannon and Cook 2015). A dominant perspective is that in historical societies battered by high death rates, the frequency of the experience, and the corresponding local norms, perceptions, practices, and beliefs that encased it, reduced the social and psychological significance of child death for parents by encouraging them to remain psychologically distant from their children (Cannon and Cook 2015). When describing premodern societies, social historians often depict bereaved parents as responding to their child’s death with indifference, casting child death as an artifact of parents’ reproduction—an unfortunate, yet not especially consequential, event. For example, in his sweeping account of the evolving conceptions of children and childhood, French historian Ariès (1962) characterized European parents during the premodern era as aloof to the death of their children, which he explained as the direct and inevitable consequence of high birth and mortality rates. Ariès (1962) contended that parents anticipated the possibility of their children’s deaths and were thus steeled against such events. Social historian Shorter (1975) argued that in preindustrial European societies, parents did not experience deep grief and mourning because it would have crippled the social order given the pervasiveness of child death. Similarly, Stone (1979) argued that parents in preindustrial England limited their psychological involvement with their children to protect themselves against what were perceived to be inevitable child deaths, ultimately preserving their own health.

More recent anthropological work on child death in high-mortality settings echoes the narratives advanced by these historical accounts. For instance, Scrimshaw (1978) writes that among Ecuadorian families, “of necessity, there is an acceptance of high rates of infant mortality and an accompanying lack of a felt need to take desperate measures to save a child’s life” (392). Riessman’s (1979) account of the Fulani in Burkina Faso emphasizes parents’ fatalism and general apathy toward child death, describing it as symptomatic of the high mortality conditions in which they live and a strategy for psychological reprieve, relieving them of the “psychological burden of feeling responsible” (182). Perhaps the most widely read anthropological work advancing the ‘neglect thesis’ is Scheper-Hughes’s (1992) account of mothers in the Alto do Cruzeiro, a shantytown in northeast Brazil. Scheper-Hughes (1992) argues that local mothers navigating economic hardship and high child mortality rates experience such destitution that they respond with indifference to child death. Scheper-Hughes (1992) contends that Alto mothers neither grieve nor mourn their deceased children, but instead are insulated from “the ravaging of grief” and, if they experience any emotion, it could be best described as “relief” (420). Scrimshaw (1978) similarly concludes that in Ecuadorian communities the death of a child “causes little disturbance” (393).

Although these anthropological accounts do not claim relevance outside the local settings they pertain to, these narratives—alongside historical accounts—imply that in societies with high mortality rates, the frequency of child death leads to a numbing of its consequences for the parents who endure it. Yet, a growing number of anthropological studies of high-mortality communities challenge this conclusion. Although some anthropologists concede that selective neglect of particularly weak and sick children may occur because of resource scarcity, this does not necessarily foreshadow nor reflect the lack of sequelae of child death for parents. Written arguably as a rebuttal to Scheper-Hughes’s work, Einarsdóttir (2005) outlines the intense reactions of bereaved parents in Guinea-Bissau despite the pervasiveness of child loss. Dettwyler (2013) similarly describes how bereaved Malian mothers become psychologically and physically distressed when recounting their deceased children. Knauft (2016) emphasizes how mothers in Papua New Guinea, where 38 percent of Gebsui children die within the first year of life, openly weep when they lose their children. Onarheim and colleagues (2017) and Sisay and colleagues (2014) write of bereaved mothers’ adverse psychological reactions in Ethiopia, where child death rates are exceedingly high. Medical anthropologist Eisenbruch (1984) argues that any notion that child death is simply met with equanimity by parents where mortality is high is “a tendentious distortion of a complex human reality” (293). Sered (1996) extends this stance and argues that such accounts indicate that “Some Westerners may find it comfortable to believe that Third World women do not love their children as we do; that African and Asian women do not care as much as we do when their children suffer and die” despite there being “…no evidence in the ethnographic record to support such a belief” (6).

Complementing anthropological efforts to render visible bereaved parents’ affliction even where child death is ubiquitous, other anthropologists emphasize the logical flaw in work that draws conclusions about bereaved parents’ psychological reactions from the study of either public mourning rituals or bereaved parents’ outward expressions. In terms of the former, some scholars contend that community rituals are not valid approximations of parents’ personal responses, and the social organization and management of death are distinct from bereavement reactions and experiences (Noret 2012). Sisay and colleagues (2014) emphasize that even though the Ethiopian context they study engages in what may be labeled as abbreviated or muted mourning rituals for a young child’s death, bereaved parents still “feel and grieve the loss” (S114).

Scholars also emphasize a parallel need to delineate theoretically between parents’ experiences, expressions, and representations of grief (Cannon and Cook 2015), suggesting parents’ psychological responses may be intense and debilitating in high-mortality settings even if not paraded publicly (Einarsdóttir 2005; M. K. Nations and Rebhun 1988; Smørholm 2016). For instance, M. K. Nations and Rebhun (1988) studied the same Brazilian community as Schepher-Hughes (1985) and argued that her focus on parents’ public reactions confounded her observations. M. K. Nations and Rebhun (1988) argue that local cultural norms, beliefs, and expectations are such that bereaved mothers often feel pressure to be outwardly stoic, even as they mourn the death of their child privately, concluding that “families care deeply for their children, and long for their survival” (191). Smørholm’s (2016) research on bereaved mothers in contemporary Zambia emphasizes how ‘silent’ grief does not equate to an absence of grief. Castle (1994) similarly describes Fulani mothers’ subdued reactions to death in Burkina Faso as often incongruous with their suffering. Matos (2007) argues pointedly that “[t]he stoic acceptance of death portrayed by Asante women is not an indicator of neglect or indifference toward their children’s death, but rather is employed as a socially sanctioned way of coping with emotional trauma” (87). In sum, these perspectives contend that even as social norms and expectations influence bereaved parents’ outward expression (Haws et al. 2010; Schut 1999), this should not be conflated with their psychological reactions to child death.

### Child death, mortality contexts, and mothers’ mental health

These accounts, combined with the vast psychology literature from low-mortality contexts, point to the possibility that bereaved parents, and in the case of our study, bereaved mothers specifically, experience measurable consequences following a child’s death, even in sociohistorical settings where child death is pervasive. In particular, a child’s death may prompt mothers to experience feelings, thoughts, and physical difficulties that are symptomatic of depression—a condition that is increasingly acknowledged locally as a public health issue in Malawi (Jassi 2024). The raw grief of a child’s death—and the mourning of what was or could have been—may directly lead to a decline in mothers’ mental health and well-being, as documented extensively in low-mortality settings (Biondi and Picardi 1996). That is, a child’s death could precipitate the hallmarks of what is understood to indicate a state of depression: distraction, disruptions to daily functioning, sadness, and guilt. The psychological consequences of a child’s death could be especially severe in a pronatal context like Malawi, where being a mother is central to women’s identity, and mothering structures women’s time and social roles (Smith-Greenaway and Yeatman 2020). As such, the premature death of a child may trigger a crisis of meaning and identity for women (Keesee, Currier, and Neimeyer 2008), leading further to their experiencing psychological and physical difficulties.

A child’s death could also precipitate a cascade of other adversities in women’s lives, such as social stigma, violence, and relationship strain (Haws et al. 2010), which could also lead to depressive symptoms. Bereaved mothers often emphasize the tremendous social and functional support they receive from close family and friends in the wake of a child’s death (Ayebare et al. 2021), but mothers can also experience blame (Castle 1994; Kiguli et al. 2016; Sisay et al. 2014). Such reactions can mean bereaved mothers experience not only social isolation and strain (Pollock et al. 2020) but also intimate partner violence (Shakespeare et al. 2019; Weitzman and Smith-Greenaway 2020) and divorce (Sisay et al. 2014), both of which can harm their mental health (Clark et al. 2020; Oram et al. 2022) and thus may further elevate their depressive symptoms.

The potential for a child’s death to influence mothers in these ways may be temporally concentrated in the initial aftermath of a death. Research on parents in the United States suggests the disruptions of bereavement often concentrate soon after the loss occurs and subside over time (M. S. Stroebe et al. 2008). Moreover, to the extent that more time elapsing since a death allows women to weather any resulting adversities, the toll of a child’s death may be greatest initially, with fewer implications as time passes. However, psychology studies provide abundant evidence of the potential for bereaved parents to experience lingering disadvantages over many years. It is thus possible that mothers in high-mortality contexts like Malawi not only experience depressive symptoms immediately following a child’s death but for multiple years to follow.

It is also possible that mothers experience depressive symptoms not only following a child’s death but simply because of the ever-present threat of child mortality. That is, in high-mortality contexts, non-bereaved parents may not be entirely unscathed by the psychological toll of child death. Some parents will have histories riddled with fearful brushes with death that may have meaningfully affected them and their family, and potentially their well-being. The ubiquitous nature of child death in high-mortality settings—combined with the fact that most child deaths result from what are otherwise routine childhood illnesses—suggests that many non-bereaved parents may have experienced the stress of a child’s illness that left them fearful they would soon become a bereaved parent. Indeed, research suggests that, on average, a child in sub-Saharan Africa experiences 15 episodes of diarrhea during the first four years of life and one to five malarial episodes per year (Boschi-Pinto, Lanata, and Black 2009; Murphy and Breman 2001). Navigating such a high burden of childhood illness against the backdrop of high child mortality rates may take a toll on parents’ mental well-being, as they are constantly reminded of the precarity of life and the ever-present threat of death (Aksan 2014).

Thus, in addition to studying experiences of child death, we also consider women’s exposure to their children’s near-death experiences, and the potential for these experiences to be independently consequential. We know little about parents’ perceptions of their children’s near-death experiences given the rarity with which they are researched. Nonetheless, studying them allows us to further test the extent to which mothers in a high-mortality setting are psychologically numb to child death—and the lurking possibility of it. If there is a numbing effect of brushes with death, then near-death experiences should have no bearing on mothers’ depressive symptoms. Alternatively, in line with evidence that death scares are theoretically significant (Pochard et al. 2005), children’s near-death experiences could be uniquely difficult and traumatic events, and thus another potential source of mothers’ depressive symptoms (Shalev et al. 1998).

## Methods

### Data and sample

To analyze the linkages between experiences of child death and mothers’ depressive symptoms, we use data from a long-running cohort study in Balaka, Malawi, Tsogolo la Thanzi (Yeatman et al. 2019). Tsogolo la Thanzi began in 2009 through the enrollment of a simple random sample of young women in Balaka, Malawi, a community in the country’s southern region. We use data from the first and third phases of the study. We begin by analyzing data from the most recent, third phase. Tsogolo la Thanzi-3 extended earlier data collection efforts by enrolling a new, population-based sample of 21- to 35-year-old women in Balaka. In June and July of 2019, interviewers completed a full household census of Balaka to enumerate the seven-kilometer catchment area. From the simple random sample of 1800 women, 1538 women completed interviews.^1^ For the purposes of this study, we excluded 122 women who had never given birth, allowing us to assess the implications of child death experiences among the sample of women at risk of such experiences.

The Tsogolo la Thanzi-3 data feature detailed information on women’s mental health, including measures of their recent depressive symptoms. The DHS Program data—the preeminent source of information on women’s and children’s health in sub-Saharan Africa—recently introduced measures of women’s self-rated health into surveys in select countries; however, no surveys feature measures of women’s psychological well-being. The Tsogolo la Thanzi-3 data thus provide an unparalleled opportunity to examine how experiences with child death, including actual deaths and near-death situations, correspond with women’s subsequent depressive symptoms. Moreover, the study collected data on other bereavement experiences and additional stressors that are likely to influence women’s depressive symptoms, further facilitating a robust analysis of the psychological toll of child death.

Even so, the cross-sectional nature of the Tsogolo La Thanzi-3 data means that unobserved factors could drive both women’s exposure to child death experiences and their depressive symptoms. Said differently, any observed associations could be due to other, unmeasured factors corresponding with both women’s propensity to experience a child’s death and depressive symptoms. Thus, as a second set of analyses, we leverage data from an earlier longitudinal study component (Tsogolo La Thanzi-1) that features closely spaced interview data, collected at four-month intervals.^2^ These data allow us to leverage time-varying information on 1490 women’s exposure to child death, as well as measures of their depressive symptoms, to explore whether changes in the former affect changes in the latter. We cannot study near-death experiences with these data but instead use repeated observations to analyze if a child’s death corresponds with a change in women’s depressive symptoms.^3^

Although child deaths are not numerous enough to study between-wave changes in women’s mental health as a function of changes in their bereavement status over the very brief four-month intervals, we still take a temporally concentrated approach to study a one calendar-year period (2009–2010) using data collected during four interviews over a 12-month period. This allows us to fully maximize our within-person design and to avoid the concern that unmeasured time-varying factors bias the results. Moreover, time-varying data on numerous stressors allow us to account for other developments in women’s lives over the years that could drive a spurious association between a child’s death and the onset, or worsening, of depressive symptoms.

### Cross-sectional analysis (2019): Measurement and statistical modeling

In the Tsogolo la Thanzi-3 phase of data collection, interviewers administered the Patient Health Questionnaire (PHQ-9) depression module to study respondents. The PHQ-9 is a self-report version of the PRIME-MD diagnostic instrument (Kroenke et al. 2010). Although this measure was not created with Malawians in mind, it has been adapted and validated in the Malawian context (Udedi et al. 2019).^4^ The PHQ-9 includes nine questions regarding whether a respondent has been bothered during the past two weeks by the following items: (1) little interest or pleasure in doing things; (2) feeling down, depressed, or hopeless; (3) trouble going or staying asleep; (4) feeling tired; (5) poor appetite; (6) feeling bad about yourself—or that you are a failure or have let yourself or your family down; (7) trouble concentrating on things; (8) moving or speaking slowly; and (9) thoughts that you would be better off not alive. Respondents reported whether they experienced these psychological and physical symptoms not at all (=0), some days (=1), more than half the days (=2), or nearly every day (=3). Following convention, we computed an overall measure of the intensity of depressive symptoms by summing responses.^5^

The Tsogolo la Thanzi-3 study also collected detailed birth and pregnancy histories from women. With these data, we can identify the subsample of women who have experienced the death of one or more children following a live birth, allowing us to create a dichotomous measure differentiating women who have (=1) versus have not (=0) experienced a child’s death.^6^ We do not restrict the measure based on child’s age at the time of death, given our relatively small sample size; instead, we focus on any parental bereavement experiences. Nonetheless, given the relatively young age profile of mothers, and the higher mortality risks in infancy and toddlerhood relative to adolescence, most of the child deaths in our data are children under age 5. In supplementary analyses, we find no evidence that having lost multiple children corresponds with distinct consequences for women’s depressive symptoms; however, the small number (*N* = 16) of multiply bereaved women in our data limits our statistical power to meaningfully explore this. Thus, we collapse any child loss experiences into a binary indicator and consider whether mothers had experienced one or more child deaths.

Women were also asked to report the year their child died. These data allow us to consider not only the consequences of having ever experienced child loss but to differentiate between deaths that happened closer to the time of the survey—and the measurement of women’s depressive symptoms—versus longer ago. Thus, in addition to considering any bereavement, we created a four-categorical measure for whether the mother experienced a child’s death within the past 3 years, 4–9 years ago, more than 10 years ago, or never. For the 16 women in the sample who had experienced two or more child deaths, we coded the timing of the child’s death according to their most recent loss.

Recognizing the pervasiveness of child death in this setting, and the corresponding reality that many non-bereaved mothers will have lived through severe child health scares, we also analyze whether any of a mother’s living children experienced near-death situations. Specifically, when asking women about their reproductive history, interviewers asked whether each of their living children had ever been close to death. We create a binary indicator to assess the ubiquitous nature of child death and whether brushes with it are associated with women’s depressive symptoms.^7^

Our models include several time-invariant, sociodemographic controls to ensure the robustness of the key associations. Given inequalities between ethnic groups in Malawi (Gondwe et al. 2021), we include respondents’ ethnicity, as well as a measure of their highest level of education completed (primary [standard], secondary [form], or tertiary) and their marital status at the time of the survey (never married, previously married [i.e., separated, divorced, and/or widowed], currently married).^8^ We also include a measure of whether the respondent experienced intimate partner violence in the past year (=1). A household goods index accounts for resource inequality in women’s home environments given that resource scarcity is related to both child death (Pamuk, Fuchs, and Lutz 2011) and women’s mental health (Mutahi et al. 2022). To do so, we constructed a household asset index derived from a principal component analysis (Smith-Greenaway and Yeatman 2020). The linear asset index comprises nine durable goods (a bed with a mattress, a television, a radio, a landline or mobile phone, a refrigerator, a bicycle, a motorcycle, an animal-drawn cart, and an automobile) and one household asset (electricity). A principal components analysis calculates weights following the same procedure used to construct the DHS Program data wealth index. The resulting index places households on a continuous scale relative to the sample. We include a measure of women’s current employment status (not employed, piece work, temporary employment, or steady job). We also include a measure for whether the respondent had lost their mother and father and their mother’s highest level of education attended (no education, primary [standard], secondary [form], or tertiary), both of which differentiate women based on their access to natal family support and resources.

To further account for other potential sources of poor health, we include a measure of whether the respondent ever experienced pregnancy loss. We also account for their number of living children, their perceived likelihood of currently being HIV positive, which is relevant to well-being in Malawi (Kohler et al. 2017), and their age (in years). We include a measure of women’s self-rated health. Interviewers asked respondents, “In general, would you say your health now is excellent, very good, good, fair, or poor.” We differentiate between women who report negative valences (fair or poor health) (=0) versus positive ones (good, very good, or excellent) (=1) (Yeatman and Smith-Greenaway 2021).

Given that adverse events are likely to affect women’s depressive symptoms, all models include additional indicators of recent negative shocks, including additional sources of bereavement. We use a series of questions asking respondents whether they experienced several stressors in the past four months, including health shocks such as whether the respondent has had health decline, has been affected by witchcraft, has had a bout of malaria, or has been hospitalized. Additionally, we use information on the health and well-being of others in their lives, including whether respondents experienced a family member’s illness. Given the potential for other deaths in respondents’ households and social networks to influence their depressive symptoms, we account for whether women experienced the death of an immediate family member (parent or sibling), close friend, or household member. To further account for additional deaths in respondents’ social networks, we include a measure of the number of funerals respondents attended in the past month. To account for whether women have been affected by economic shocks, we include whether they experienced a shortage of food or lost their jobs/transitioned to a worse job. Finally, to account for recent relationship stress, we include an indicator for whether the respondent experienced partner infidelity.

In the main results, we model the composite PHQ-9 depression score continuously, and thus we estimate ordinary least squares regression models. Supplementing these results, we fit additional models using other measures of depressive symptoms, including the presence of “severe” depressive symptoms (per the PHQ-9), as well as alternative, self-reported measures of recent depressive feelings (see Online Appendices Tables 1–3), each of which are tailored to the distribution of the outcome variable.

### Longitudinal analyses (2009–2010): Measurement and modeling

Whereas the Tsogolo la Thanzi-3 cross section allows us to study women’s cumulative exposure to child death, with further consideration of the timing of the death and near-death experiences, the Tsogolo la Thanzi-1 data allow us to take a longitudinal approach to study the immediate consequences of a child’s death for a woman’s depressive symptoms—net of her pre-bereavement reports. The longitudinal Tsogolo la Thanzi-1 study phase did not, however, feature the PHQ-9 depressive symptom questionnaire; instead, interviewers simply asked respondents if they had felt depressed in the past month.^9^ This item is similar to an item on the PHQ-9 assessment as well as items on the Beck Depression Inventory (Kim et al. 2014) and the Perceived Stress Scale (PSS-4) (DeVylder et al. 2016), both of which have also been adapted and locally validated in Malawi. Specifically, interviewers asked respondents if, in the past month, they felt not really, a little, or very much depressed, which we collapse into a binary for whether women felt very depressed. In models of this outcome, we consider women’s time-2 response (which, for some women, was after having experienced a child death) in relation to their time-1 response.

At waves 2, 3, and 4, interviewers asked women if they had experienced a child’s death since their last interview; during the full 12-month period of the study covered by these four data waves, 2 percent of women experienced a child’s death. This allows us to assess if—over the course of a one-year study period—bereaved women have distinct odds of experiencing an increase in depressive feelings (relative to their pre-bereavement response) compared to their non-bereaved peers—net of a rich set of time-varying factors.

Specifically, we estimate a series of linear probability models to examine if a change in women’s status as a bereaved mother corresponds with a change in their probability of feeling very depressed. This model allows us to establish the percentage increase in the probability of a woman developing depressive feelings between waves 1 and 4 (a 12-month period) as a function of a child’s death. A major advantage of this modeling approach is that it accounts for all time-invariant characteristics. Changes in depressive feelings can, however, be driven by time-varying factors. As such, in these models, we use the same measures of negative shocks as described above and included in the cross-sectional Tsogolo la Thanzi -3 models.^10^

## Results

### Descriptive results

We begin by describing the Tsogolo la Thanzi-3 data (2019), which we use to estimate cross-sectional associations between experiencing a child’s death and/or near-death experiences and women’s depressive symptoms. Table 1 provides descriptive statistics to characterize the sample. On average, respondents have a PHQ-9 depressive symptom score of 5, reflecting what is generally considered indicative of mild depression—although this calibration is not specific to the Malawian context.

Table 1 further shows that about one-in-six women (13.8 percent) experienced a child’s death at some point in their lives—a staggering estimate given the sample’s young age profile (ages 21–35 years). Just 3.0 percent experienced a death in the past three years, 3.7 percent experienced a child’s death four to nine years earlier, and 7.1 percent experienced a child’s death longer ago. Equally striking is that 44.1 percent of women reported that at least one of their surviving children had a near-death experience, emphasizing the palpability of child death in this context.

Table 1 further shows that the ethnically diverse sample had mostly discontinued school at the primary level; approximately one-third attended secondary school. Most respondents were currently married (77.8 percent), and were, on average, 27 years old. Just over one-half (50.2 percent) of respondents had lost both their parents, and nearly one in five (18.5 percent) reported fair or poor health. The results show the high burden of adverse events that transpired in women’s lives in the four months prior to the survey, emphasizing the uncertainty that defines life in this context. Nearly one-in-six women (13.4 percent) had recently experienced a decline in health, 24.2 percent had malaria, and 10.1 percent were hospitalized. More than two-thirds of women had experienced illness in their family, and 18.2 percent had recently experienced the death of a close friend, relative, or household member. Women had, on average, attended 1.3 funerals in the past month (ranging from 0 to 8; with 64 percent having recently attended at least one funeral)—showing the steady presence of death that Malawian women endure in their families, households, and communities. The results further show the high rate of economic stress on women: 62.2 percent experienced a shortage of food recently.

Table 1 characterizes the groups of women who had and had not experienced a child’s death, clarifying similarities and differences in their profiles. Bereaved mothers have significantly more depressive symptoms relative to their non-bereaved peers (6.2 vs. 4.8, *p* < 0.001). The sociodemographic profiles of women who experienced a child’s death tend to be disadvantaged relative to those who have not—emphasizing child loss as itself a marker of hardship. Bereaved mothers tend to be less educated, single, and poorer relative to their non-bereaved peers, and they are more likely to be orphaned. The social patterning of child loss in this context attests to the need to ensure any evidence of inequality in depressive symptoms by women’s bereavement status is not attributable to these overlaying inequalities.

Comparably high percentages of women report a surviving child had a near-death experience (43.9 percent of bereaved women and 44.2 percent of never-bereaved women)—evincing the pervasiveness of the threat of child death in this setting. Moreover, the results show the generally equal burden of recent hardships experienced by bereaved and non-bereaved women. Although bereaved women are slightly more likely than their non-bereaved peers to have recently experienced food shortage, familial illness (perhaps attributable to their deceased child), and partner infidelity, comparable percentages of women had experienced each of the numerous other negative health and economic shocks, as well as other forms of bereavement, emphasizing the precarity faced by all women in Balaka.

### Child death, the threat of child death, and mothers’ subsequent depressive symptoms

Turning to the multivariable analysis of the cross-sectional data, Table 2 presents results of the regression analyses exploring the associations between child loss and depressive symptoms. As shown in Model 1 of Table 2, having experienced a child’s death corresponds with women having significantly more depressive symptoms (*p* < 0.01). This counters the idea that women are psychologically numb to child death given its pervasiveness.

**FIGURE 2:**
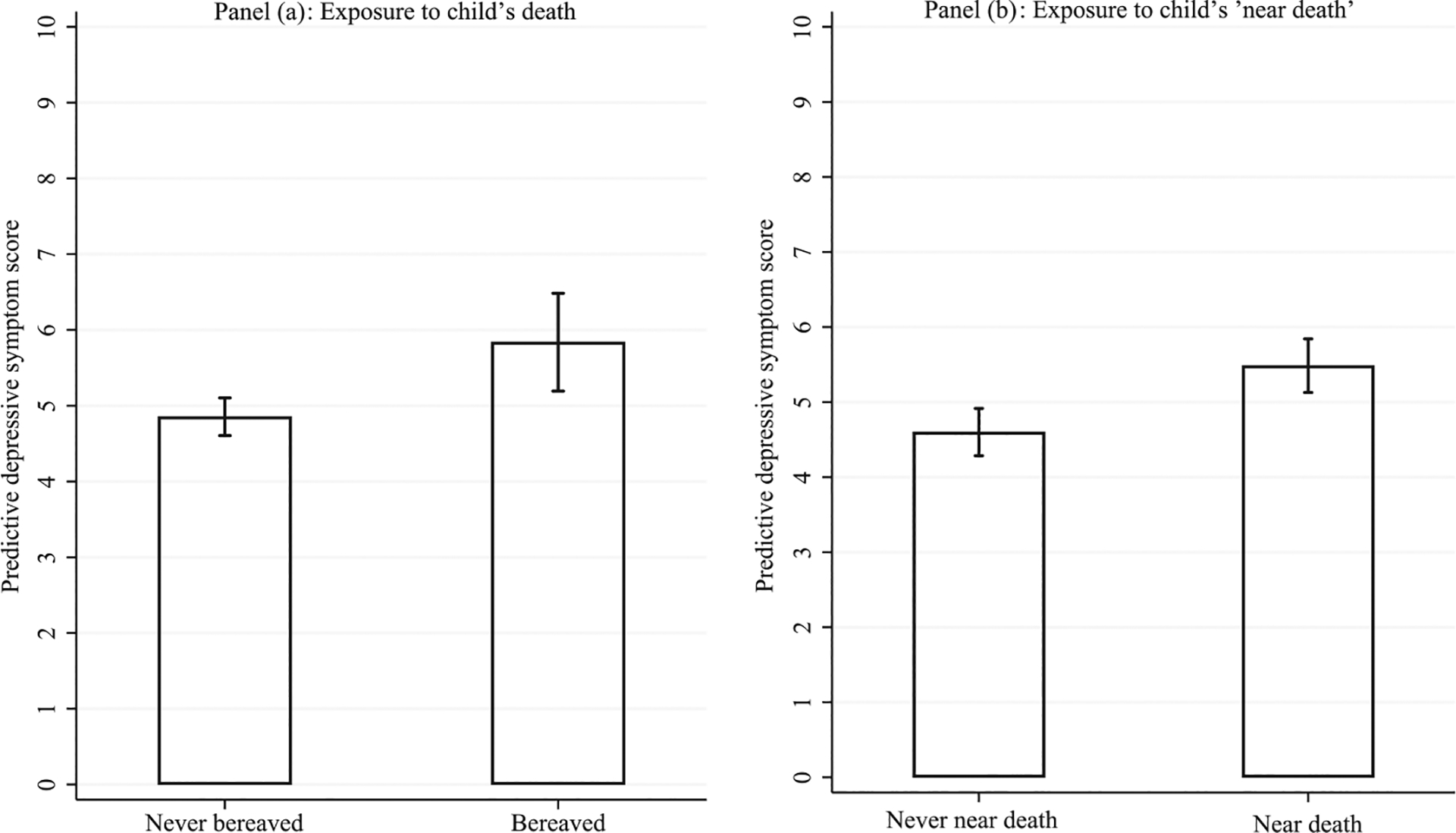
Predictive depressive symptom scores by bereavement status among women in Balaka, Malawi, enrolled in the Tsogolo la Thanzi-3 study NOTE: Results estimated using the average marginal effects; based on results shown in Table 2.

To quantify the magnitude of this significant association, panel a of Figure 2 depicts the predictive depressive symptom scores for bereaved and never bereaved women while holding constant all covariates shown in Model 1 of Table 2. Bereaved mothers have a predictive depressive symptom score of 6, whereas women who are not bereaved have a predictive depressive symptom score of 5. Given that 90 percent of the sample score lower than 11 on the depressive symptom score (median score = 4 points), a one-point difference signifies the notable disadvantage associated with bereavement.

Above and beyond the consequence of mothers’ bereavement, surviving a child’s near-death experience also corresponds with more depressive symptoms. The magnitude of this association is depicted in panel b of Figure 2: women who report never experiencing a child’s near death have a predicted depressive symptom score of 4.5, whereas those who have experienced a child’s near death have a predicted depressive symptom score of 5.5—again representing a sizeable difference between women.

Model 1 of Table 2 further shows that although sociodemographic factors are not, in general, associated with women’s depressive symptoms, aside from select evidence of small ethnic group differences (e.g., Tonga ethnicity corresponds with significantly higher depressive symptoms), women who had a pregnancy loss, experienced intimate partner violence, and have poor self-rated health have more depressive symptoms. Recent negative shocks are, however, more consistently associated with women’s depressive symptoms. Familial illness and death also correspond with more depressive symptoms, emphasizing the salience of other deaths in women’s families, households, and social circles.

Given evidence that child death corresponds with mothers’ depressive symptoms net of a host of other correlates, we next examine whether these associations are driven by more recent child deaths or also stem from deaths that happened several years prior. Model 2 of Table 2 shows the results disaggregated by whether the loss occurred within the past few years (≤3 years), several years ago (4–9 years), or at least a decade ago. Mothers who experienced a child’s death a decade or longer ago do not have significantly more depressive symptoms than their non-bereaved peers. Instead, in alignment with the broader psychology literature on bereavement, the results show that more recently bereaved mothers tend to have more depressive symptoms. Additional analyses (not shown) demonstrate that recently bereaved women have predictive depressive symptom scores approaching 7, placing them in the 75th percentile. Mothers who experienced a death even several years ago (i.e., 4–9 years) have more symptoms relative to their non-bereaved peers; however, the association is marginally significant (*p* < 0.1).

### Robustness checks

#### Measurement of depressive symptoms.

The above results firmly establish a relationship between child loss and mothers’ depressive symptoms, but they focus only on a continuous measure of symptoms, offering no indication that bereavement is tied to especially severe outcomes. Thus, in additional analyses, we recode the PHQ depressive symptoms score to denote whether women are among the 4.7 percent of the sample who have scores that convention establishes constitute severe depression (PHQ score = 15+). As shown in Model 1 of Online Table A1, having experienced a child’s death corresponds with 2.40 times the odds of experiencing severe depressive symptoms *(p* < 0.05). Model 2 of Online Table A1 further shows that the odds of severe depressive symptoms are highest for women bereaved in recent years: women who experienced a child’s death during the past three years have 3.51 times the odds of experiencing severe depression relative to never-bereaved women (*p* < 0.05). Women who experienced a child’s death even several years ago, however, continue to have an elevated risk of severe depression: women whose child died four to nine years ago experience 2.98 times the odds of severe depression relative to never-bereaved women (*p* < 0.05). Again, the results suggest depressive symptoms will be more likely not only for bereaved mothers but also for mothers who consider themselves to have been almost bereaved: having a child near death corresponds with a 69 percent increase in the odds of severe depressive symptoms (odds ratio: 1.69, *p* < 0.1); however, notably the association is of marginal significance.

Although the PHQ has been adapted and validated in the context of Malawi, we are not aware of its validation among postpartum mothers—which applies to some of the women in our sample. Moreover, some of the physical symptoms of depression included in the PHQ, notably feeling tired and difficulty sleeping, are symptoms of other common diseases (e.g., malaria) and underlying conditions (e.g., anemia) in Malawi. The PHQ has also been contested in terms of questions regarding suicidology (Chung et al. 2023). Thus, before turning to the TLT-1 results, we performed a series of supplementary analyses to confirm the robustness of the associations to an alternative, self-reported measure of the frequency by which women “felt depressed” in the prior month. This question was asked in Chichewa using common, conversational phrasing and thus is arguably a more localized approach to soliciting information about women’s mental health.

We code women’s depressive feelings both ordinally and as a binary indicator to denote having felt depressed often. Beginning with the ordinal coding, as shown in Models 1 and 2 of Online Table A2, although having ever experienced a child’s death is not significantly associated with the frequency by which women felt depressed in the month prior to the survey, having experienced a child’s death in the past three years corresponds with2.32 times the odds of feeling depressed more frequently (*p* < 0.05). The results further show that a child’s near death corresponds with a 47 percent increase in the odds of women’s more frequent depressive feelings (odds ratio: 1.47, *p* < 0.01). Online Table A3 shows that these findings are replicated when we recode recent depressive feelings as a binary indicator to distinguish the 11.9 percent of the sample who report having felt depressed often. Model 2 in Online Table A3 demonstrates that experiencing a child’s near death corresponds with 1.76 times the odds of feeling depressed often (*p* < 0.01) and having experienced a child’s death in the prior three years corresponds with 3.18 times the odds of feeling depressed often (*p* < 0.05).

#### Changes in mothers’ depressive feelings following child death.

To further assess the robustness of these cross-sectional associations, we present linear probability models that use repeated measures of women’s self-reported depressive feelings, collected in Tsogolo la Thanzi-1, as well as data on women’s experiences of child death during the one-year focal period. Before turning to the multivariable models, which assess if a change in women’s bereavement status corresponds with a change in their feeling very depressed, Table 3 reports women’s responses at the time of the baseline interview and the follow-up interview one year later, separately for women who experienced a child’s death over the study period and those who did not. Women who experienced a child’s death during the year are much more likely to feel very depressed at the one-year mark relative to women who did not experience a child’s death, even though they were less likely to feel depressed at the baseline interview. Specifically, at the end of the observation year, only 7.4 percent of non-bereaved mothers felt very depressed in the prior month, compared to 30.4 percent of bereaved mothers. When considering individuals’ own trajectory relative to their baseline report, 13.7 percent of non-bereaved women experienced more intense depressive feelings over the study year, compared to 39.1 percent of bereaved women.

To assess whether these descriptive differences persist in a multivariable framework, Table 4 presents linear probability models, which hold constant all time-invariant factors and account for a host of time-varying covariates. As shown in Table 4, having experienced a child’s death during the year corresponds with a 25 percent increase in the probability of feeling very depressed at the one-year mark—even after accounting for other negative health and economic shocks. In conjunction with the cross-sectional results, these findings emphasize the measurable consequences of child death for mothers in a setting where child death—and the threat of it—are ubiquitous.

## Discussion

This study contributes to the extant demographic literature on child death, as well as broader debates spanning anthropology, history, and psychology about the effects of child death on parents. We highlighted the heavy burden of child death that parents continue to navigate in contemporary Malawi. As recently as 2015, DHS Program data show that reproductive age mothers had a 40 percent probability of experiencing a child’s death before age 50. This heavy burden is replicated in the Tsogolo la Thanzi-3 data, wherein upward of one in six young mothers in their 20s and early 30s had already experienced a child’s death. These estimates emphasize that child loss continues to be a normative feature of motherhood—despite the precipitous declines in under-five mortality rates in Malawi. Beyond their own children, the study demonstrates the immense loss women navigate in their broader familial and social networks: one-in-five women had experienced the death of a close relative in the few months preceding the survey, and recent funeral attendance was nearly universal.

The study also shows that—even amid the pervasiveness of death in this context—experiencing the death of a child comes with measurable consequences for mothers. A child’s death significantly increases women’s depressive symptoms in the shorter and medium terms, with additional evidence that a death can precipitate the onset of severe depressive feelings. The imprint of a young child’s death on mothers is most clearly observed in the first few years after the death, but, in some cases, bereaved mothers’ elevated likelihood of depressive symptoms does not become statistically indistinguishable from their non-bereaved peers for as long as a decade. Given the long-lasting effects a death has on mothers’ well-being, combined with the consequential nature of other sources of bereavement in women’s families and communities, the results imply that the constant onslaught of losses does not numb women to a child’s death; rather, a child’s death thrusts mothers into a “constant state of mourning” (Millward 2016, 162). Together, these findings defy the notion that child death is met with equanimity and resignation simply because it is a frequent parental experience. Instead, the robust, population-based evidence of the toll of bereavement emphasizes the anguish mothers experience in the wake of child loss—with additional evidence that pregnancy loss is also consequential—a subject that has been overlooked in studies of African women’s well-being (Palfreyman and Gazeley 2022). In doing so, the results offer foundational insights into underappreciated reproductive-related sources of women’s health disparities.

Although the “neglect thesis,” which our results refute, has been most widely adopted by anthropologists and social historians, and not explicitly advanced by demographers, our findings emphasize the need to expand the demographic canon in notable ways. Demographic research has arguably shown more interest in highlighting parents as active participants in their children’s passing rather than as secondary victims of their children’s demise. Demographic scholarship has emphasized how parental preferences (Chen, Huq, and d’Souza 1981; Levine 1987) and desires (Singh, Singh, and Mahapatra 2013), as well as parenting (Das Gupta 1990), can affect children’s risk of death. Our results attest to the need for future population research to acknowledge child death not only as an ad verse outcome—an indicator of a life cut short—but also as a demographic event that has measurable consequences for the proximal others who endure it.

The results demonstrate that even brushes with child death can take a toll on women, further challenging the notion of preemptive neglect or numbing. Nearly one-half of mothers reported that at least one of their surviving children had a near-death experience—highlighting the lurking threat of death that Malawian parents navigate. Yet, the pervasiveness of child death does not mean its occurrence is inconsequential; rather, the ever-present threat of child death wears not only on the parents who experience it but also on parents who come close to experiencing it. Having a child near death corresponds with mothers’ significantly higher depressive symptoms—regardless of whether they ever actually experienced a child’s death.

By demonstrating the theoretical salience of both actual and near-death experiences, the results raise questions of the possible interplay between the two, including the potential for death scares to affect how women react to a subsequent child’s death, and vice versa, for a child’s death to alter women’s experience of subsequent “close calls.” In terms of the former, having one or more near-death experiences precede a child’s death could amplify the consequences of the eventual death, as stressful experiences tend to accumulate. Or, in alignment with the numbing hypothesis, such experiences could ultimately lessen the blow of a death. It could be especially difficult for a bereaved mother to navigate a subsequent death scare, or she may face it with aplomb, being less susceptible to the fear of what has already come to pass. Unfortunately, we only have data on whether mothers have endured a child’s near-death experience; we lack information on the timing of such experiences, and thus their sequencing in relation to actual child deaths, thereby prohibiting us from ascertaining any interplay between mothers’ reactions to each. Nonetheless, by demonstrating the simultaneous influence of both near-death experiences and actual child losses on mothers, the results emphasize the cumulative effect of these experiences, offering an even more disconcerting account of the adverse effects that the constant threat of child death poses to women in Malawi, and possibly other mortality burdened settings.

Our study breaks new ground by documenting the significance of children’s near-death experiences for mothers’ well-being, but the very novelty of this measure limits it. Mothers’ recollections or framings of what constitutes a near-death experience are likely to vary widely and may not always accurately represent the objective severity of a health scare. That is, a mother’s memory of a child being close to death may be exaggerated relative to a health provider’s assessment, or mothers may fail to acknowledge real brushes with death that stemmed from situations for which they felt culpable. Even so, growing recognition that individuals’ perceptions of experiences and realities, not just objective measures of them, are of demographic relevance across a broad swath of life domains (Glei, Goldman, and Weinstein 2018) supports the logic and utility of this measure. Indeed, the significant associations between near-death experiences and women’s depressive symptoms support further investigation into how to theorize and measure perceptions of child death in future work.

Our focus on a singular dimension of women’s well-being—depression—also limits the study. We conducted a number of robustness checks to examine the stability of the results to various measures pertaining to depressive feelings and symptoms, and thus we offer a rigorous analysis of this particular aspect of women’s psychological well-being, which is viewed locally as a real and consequential health issue facing Malawian adults. Yet, calls for the global health field to expand beyond its conventional depression-centric framework are mounting, given, among other issues, the fact it is steeped in a Western biomedical approach. Thus, future efforts to take a more comprehensive approach to studying the consequences of child loss on mothers’ psychological and emotional well-being are critically important (Kathono et al. 2024; Mhango, Michelson, and Gaysina 2023; Palfreyman and Gazeley 2022). Such efforts should strive to meaningfully calibrate individuals’ emotional and psychological health and well-being in locally salient ways. While we use an imported, validated symptom screener tool, the PHQ-9, we also show that the findings replicate when relying on a simple question asking women how they have felt recently—a question asked of them conversationally using colloquial terms. As demographers increasingly venture into new intellectual terrain to study mental health and psychological well-being as historically overlooked dimensions of population health, it is a propitious time for the field to also contribute to ongoing debates about their measurement.

Measurement concerns aside, the study is also limited in other ways that leave much for future work. We focused on mothers, excluding fathers due to the small number of men enrolled in the Tsogolo la Thanzi study, raising questions of whether the current findings extend to men. Anthropological evidence of the salience of child death to fathers is conflicting. Some accounts emphasize bereaved mothers’ stronger reactions (see, e.g., Spring’s [1978] research on Zambian families and Finkler’s [1985] study of Mexican families)—a narrative echoed by the psychology literature focused on contemporary North American and Western European families (Dyregrov and Matthiesen 1987). Other scholars, however, emphasize the toll that child loss has on fathers (see, e.g., Einarsdóttir’s [2005] research on families in Guinea-Bissau and M. K. Nations and Rebhun’s [1988] research on Brazilian families). Incorporating fathers in research related to child loss will help clarify the full extent of its health consequences for adult populations. The results also pertain only to the Malawian context, laying the groundwork for future efforts to explore the implications of bereavement for adults’ health and well-being in other areas where child death remains widespread. Although there are few empirical studies available at present, select work implies these findings may translate to other contexts and other health domains (Smith-Greenaway, Yeatman, and Chilungo 2022; Weitzman and Smith-Greenaway 2020).

Finally, the study does not attend to possible axes of variation in the associations, specifically circumstantial factors like the cause or timing of the death in relation to the child’s or mother’s life. Due to our sample size, and the resulting small cell sizes, we are not well positioned to parse possible within-population differences in women’s reactions to variable experiences of child loss. Anthropologists have long emphasized between-society differences in reactions to child death, but exploring empirically within-society differences may also prove fruitful.

Leaving much for future research, our findings have practical and theoretical import. A sizeable population of parents stands to benefit from efforts to incorporate parental bereavement support into policies and programs (McNeil et al. 2020). Efforts to prevent child death in the first place are essential, especially given that the deprivation that puts women at risk of child loss is as much implicated in their poor mental health as the bereavement experience itself. Yet, families also need community-based, programmatic efforts to lessen the consequences when a child does die. This is not to suggest the grief-induced sequalae we document here can be erased; yet efforts to support bereaved parents may benefit not only their psychological well-being but also other dimensions of their, and their surviving children’s, lives.

More broadly, our results stand to reorient population researchers’ conceptualization of the relevance of child death as not only a child health outcome but also an explicitly parental event. Demographers have been quick to turn to parents—mothers especially—as dutiful reporters of the short lives that our surveys would otherwise fail to capture. Demographers recognize that mothers are best suited to ensure these lives and deaths are recorded; yet we then reflexively revert to the treatment of child death as an event that happens only to an individual—a marker of a single life cut short. Our research highlights the problematic nature of this reflexivity, which has led the population health consequences of parental bereavement to remain invisible to demographers. Examining the consequences of child loss—and the lurking threat of it—identifies previously overlooked sources of adult health disparities in sub-Saharan Africa. In doing so, the study emphasizes the value of a paradigm shift toward further recognition of child death not only as a consequence but also as a cause of population health.

## Supplementary Material

supp material

## Figures and Tables

**TABLE 1:** Characteristics of women in Balaka, Malawi, enrolled in the Tsogolo la Thanzi-3 study

	Full sample		Never bereaved		Bereaved mothei	
Variable	Mean (SD)/%		Mean (SD)/%		Mean (SD)/%	
Depressive symptom score	5.0	(4.9)	4.8	(4.7)***	6.2	(5.4)
Ever experienced child’s death	13.8					
Timing of child’s death						
≤3 years	3.0					
4–9 years ago	3.7					
10+ years ago	7.1					
Never bereaved	86.2					
Ever experienced child’s near death	44.1		44.2		43.9	
*Sociodemographic characteristics*						
Ethnicity						
Yao	27.3		28.0		23.0	
Chewa	6.7		6.2		10.2	
Lomwe	18.1		18.9		12.8	
Tumbuka	0.9		1.0		0.5	
Ngoni	42.2		41.2		48.5	
Sena	2.2		2.2		2.0	
Tonga	0.4		0.4		0.5	
Other	2.1		2.1		2.6	
Women’s educational attainment						
Primary	63.4		61.2***		77.0	
Secondary	33.8		35.7***		21.9	
Tertiary	2.8		3.1***		1.0	
Marital status						
Currently married	77.8		78.3		75.0	
Previously married	15.5		14.6		21.4	
Never married	6.6		7.1		3.6	
Women’s age	27.5	(4.4)	27.2	(4.3)***	2.5	(1.3)
Total number of children	2.4	(1.2)	2.3	(1.2)	2.5	(1.3)
Experienced pregnancy loss	11.7		9.6***		25.0	
Household goods index	−0.4	(2.2)	−0.3	(2.2)*	−0.6	(2.2)
Employment status						
Does not work	37.6		38.4		32.6	
Piece work	12.4		12.6		11.2	
Temporary work	12.6		12.6		12.2	
Steady work	37.4		36.4		44.0	
Experienced IPV in the prior year	10.0		9.0**		16.3	
Perceived likelihood infected with HIV	2.5	(3.4)	2.5	(0.1)*	2.9	(0.3)
Fair/poor health	18.5		18.1		20.9	
Orphan	50.2		48.8**		59.2	
Mother’s education						
None	17.9		17.2		21.9	
Primary	67.2		68.3		60.7	
Secondary	8.5		8.4		9.2	
Tertiary	1.2		1.3		0.5	
Don’t know	5.2		4.8		7.7	
*Shocks* ^a^						
Health declined	13.4		13.0		16.3	
Affected by witchcraft	2.5		2.5		2.6	
Had malaria	24.2		23.8		26.5	
Hospitalized	10.1		10.2		9.7	
Food shortage	62.2		61.0*		69.4	
Lost job/worse job	3.7		3.7		3.6	
Partner infidelity	20.7		19.9*		25.5	
Family illness	67.7		66.6*		74.0	
Number of funerals attended	1.3	(1.2)	1.3	(1.2)	1.4	(1.2)
Additional bereavement	18.2		18.0		19.4	
N	1,416		1,220		196	

NOTE: Tsogolo la Thanzi-3 data

Statistical significance denotes *t*-test results;

*** *p* < 0.001;

** *p* < 0.01;

* *p* < 0.05.

a Shocks are measured within the past four months.

**TABLE 2:** Ordinary least squares regression model results of the intensity of depressive symptoms among women in Balaka, Malawi, enrolled in the Tsogolo La Thanzi-3 study

	Model 1		Model 2	
Variable	Coefficient	95% CI	Coefficient	95% CI
Ever experienced child’s death	0.98**	[0.28, 1.68]		
Timing of child’s death				
≤3 years			1.75*	[0.25, 3.26]
4–9 years ago			1.12^+^	[−0.05, 2.29]
10+ years ago			0.61	[−0.33, 1.55]
Never bereaved			–	–
Ever experienced child’s near death	0.88***	[0.39, 1.37]	0.88***	[0.38, 1.37]
*Sociodemographic characteristics*				
Ethnicity				
Yao	–	–	–	–
Chewa	−0.09	[—1.09, 0.92]	−0.07	[−1.08, 0.98]
Lomwe	0.00	[−0.72, 0.71]	0.01	[−0.71, 0.72]
Tumbuka	0.04	[−2.48, 2.56]	0.03	[−2.49, 2.55]
Ngoni	0.37	[−0.21, 0.95]	0.38	[−0.20, 0.96]
Sena	0.05	[−1.59, 1.69]	0.04	[−1.60, 1.68]
Tonga	4.29*	[0.68, 7.91]	4.27*	[0.66, 7.89]
Other	0.51	[−1.17, 2.18]	0.49	[−1.18, 2.17]
Women’s educational attainment				
Primary	–	–	–	–
Secondary	0.13	[−0.46, 0.72]	0.14	[−0.46, 0.73]
Tertiary	0.01	[−1.65, 1.68]	0.03	[−1.63, 1.70]
Marital status				
Currently married	–	–	–	–
Previously married	0.07	[−0.61, 0.75]	0.08	[−0.60, 0.76]
Never married	0.27	[−0.72, 1.26]	0.27	[−0.72, 1.26]
Experienced pregnancy loss	0.74*	[0.00, 1.48]	0.78*	[0.04, 1.52]
Total number of children	−0.09	[−0.35, 0.18]	−0.08	[−0.35, 0.19]
Household goods index	0.00	[−0.14, 0.13]	0.00	[−0.14, 0.14]
Employment status				
Does not work	–		–	
Piece work	0.49	[−0.29, 1.27]	0.49	[−0.29, 1.27]
Temporary employment	0.24	[−0.53, 1.00]	0.24	[−0.53, 1.00]
Steady employment	−0.26	[−0.82, 0.29]	−0.26	[−0.81, 0.29]
Experienced IPV in the prior year	0.95*	[0.16, 1.75]	0.96*	[0.17, 1.75]
Perceived likelihood infected with HIV	−0.05	[−0.13, 0.02]	−0.05	[−0.12, 0.02]
Women’s age	−0.02	[−0.09, 0.05]	−0.02	[−0.09, 0.06]
Fair/poor health	2.67***	[2.03, 3.31]	2.67***	[2.03, 3.31]
Orphan	−0.37	[−0.86, 0.11]	−0.37	[−0.85, 0.12]
Mother’s education				
None	–	–	–	–
Primary	0.29	[−0.34, 0.93]	0.30	[−0.34, 0.93]
Secondary	0.38	[−0.65, 1.40]	0.38	[−0.65, 1.41]
Tertiary	1.67	[−0.67, 4.01]	1.62	[−0.72, 3.96]
Don’t know	0.26	[−0.90, 1.42]	0.25	[−0.91, 1.42]
*Shocks* ^a^				
Health declined	1.49***	[0.77, 2.21]	151***	[0.79, 2.22]
Affected by witchcraft	0.11	[−1.36, 1.59]	0.15	[−1.32, 1.63]
Had malaria	0.64*	[0.09, 1.20]	0.65*	[0.09, 1.20]
Hospitalized	0.61	[−0.18, 1.41]	0.59	[−0.21, 1.38]
Food shortage	0.78**	[0.23, 1.32]	0.79**	[0.24, 1.33]
Lost job/worse job	1.48*	[0.23, 2.72]	1.43*	[0.19, 2.68]
Partner infidelity	1.02***	[0.43, 1.61]	1.03***	[0.43, 1.62]
Family illness	0.83**	[0.31, 1.36]	0.84**	[0.31, 1.36]
Number of funerals attended	0.09	[−0.10, 0.28]	0.09	[−0.10, 0.29]
Additional bereavement	1.22***	[0.61, 1.83]	1.20***	[0.58, 1.81]
*R*-squared	0.20		0.20	
*F*-test	9.19***	–	8.77***	

NOTE: Tsogolo la Thanzi-3 data; *N* = 1416. Abbreviation: CI, confidence interval.

a Shocks are measured within the past four months except for funeral attendance, which pertains to the prior month.

*** *p* < 0.001;

** *p* < 0.01;

* *p* < 0.05;

+ *p* < 0.1.

**TABLE 3:** Descriptive statistics of mothers’ experiences of child death and depressive symptoms in Balaka, Malawi, enrolled in Tsogolo La Thanzi-1

	Full sample (%)	Never bereaved (%)	Bereaved (%)
	100.0	98.0	2.0
Baseline depression			
Not very depressed	92.5	92.3	100.0
Very depressed	7.6	7.7	0.0
Depression one year later			
Not very depressed	92.2	92.6	69.6
Very depressed	7.8	7.4	30.4
*More depressed over one year*	14.2	13.7	39.1

NOTE: Tsogolo la Thanzi-1 data; *N* = 1490.

**TABLE 4:** Linear probability models estimating the likelihood of reporting feeling ‘very much’ depressed among women in Balaka, Malawi, enrolled in the Tsogolo La Thanzi-3 study

Variable	Coefficient	SE
Child death in the past year	0.25**	0.07
Health declined	0.11**	0.04
Affected by witchcraft	−0.21^+^	0.11
Had malaria	−0.03^+^	0.02
Hospitalized	0.12*	0.05
Food shortage	0.00	0.03
Lost job/worse job	−0.43**	0.15
Partner infidelity	0.14**	0.05
Married	0.02	0.04
Divorced	0.11	0.07
Had baby	0.01	0.05
Became pregnant	−0.05	0.04
Family member illness	0.02	0.02
Household bereavement	0.10*	0.04
Number of funerals attended	0.00	0.01
F-test	5.31***	
Rho	0.44	

NOTE: Tsogolo La Thanzi -1 data; *N* = 1490 women (2824 observations). Abbreviation: SE, standard error.

*** *p* < 0.001;

** *p* < 0.01;

* *p* < 0.05;

+ *p* < 0.1.

## Footnotes

1 Among the sample, 81 women were not eligible because listing data suggested they met the age/gender restriction, but they did not; 110 women migrated before the study; 42 could not be located; 21 refused to participate; and 8 had physical/mental impairment that prohibited participation. Note that these women—particularly those who could not be located, refused to participate, or were impaired—may differ from participating women in terms of their bereavement experiences and depressive symptoms. Given we lack data on these women, we are not able to explore the extent of this possibility; like all observational studies, selective participation has the potential to bias the sample characteristics. Although we do not anticipate this will systematically shift the key associations shown here, it may lead to more conservative estimates of the prevalence of bereavement and depressive symptoms.

2 We use the data available for public use through the Inter-university Consortium for Political and Social Research Data Sharing for Demographic Research (DSDR): https://www.icpsr.umich.edu/web/DSDR/series/767. The Eunice Kennedy Shriver National Institute of Child Health and Human Development funds DSDR under grant R24 HD048404.

3 This earlier phase of Tsogolo la Thanzi also features data on a simple random sample of men; however, owing to the small sample size, a mere six men reported a child’s death during Tsogolo la Thanzi-1, hence our focus exclusively on women.

4 Given potential shortcomings of this screener tool, we also explore the robustness of all key results to more global, self-reported measures of women’s depressive feelings.

5 Following guidelines for severity of depressive symptoms, in supplementary analyses we model whether child death corresponds with meeting the threshold of “severe” depressive symptoms (coded as none to moderate depression [0–14] vs. severe depression [15–27]).

6 Given the known issues with differentiating between stillbirths and early neonatal deaths (Haws et al. 2010), some of the child loss experiences reported here may pertain to late fetal losses. 73 women reported they experienced a stillbirth in a separate pregnancy history module, and 36 of these women (49 percent) reported having lost a child. As such, while it is possible that a small number of women coded as having lost a child are reporting on a stillborn child, at most, it would represent a small proportion of the child death experiences studied here. Note that we also include a measure of pregnancy loss as a covariate.

7 The majority (71.5 percent) of women who endured a child’s near-death experience reported only one child having such an experience; 28.5 percent of women reported that at least two of their children had been near death. In supplementary analyses, we explored whether the latter women experienced worse depressive symptoms than those who experienced only one child’s near death; we found no statistical evidence that this was the case.

8 In the Malawian context many marriages are informal (Bertrand-Dansereau and Clark 2016); “currently married” thus includes women living informally with a romantic partner. In supplementary analyses, we maintained separate categories for separated, divorced, or widowed women. The null linkages between previous marriage and depressive symptoms replicated, perhaps owing to the small percentage of women in each category.

9 Specifically, interviewers asked women in Chichewa: “Munali okhumudwa.”

10 All shocks are coded as = 0 at the time of the 2009 baseline survey, given that shocks data were not collected at this interview. We then consider whether women experienced a negative health or economic shock using their wave-4 reports (measured a year after the baseline study). In supplementary models, we revised our coding of these time-varying characteristics to = 1 if women reported any experience of a negative shock at waves 2, 3, or 4—not wave 4 alone. Using this more cumulative approach to measure other adversities in women’s lives over the focal one-year study period, rather than only the wave 4 measure, produced results nearly identical to those we present here.
