# Supplementary material for: Child Death and Mothers’ Subsequent Mental Health in a High-Mortality African Community
Source: Popul Dev Rev. Author manuscript; Available in PMC 2025 Dec 1. (PMC11737056; doi:10.1111/padr.12682)
Supplement: supp material [file NIHMS2045393-supplement-supp_material.docx]

**Appendix: Supplemental Materials**

| TABLE A1 Logistic regression model results of severe depressive symptom scores using the Patient Health Questionnaire-9 depression module among women in Balaka, Malawi, enrolled in the Tsogolo La Thanzi-3 study, expressed as odds ratios | | | | | | | |
| --- | --- | --- | --- | --- | --- | --- | --- |
| Variable |  |  |  |  |  |  |  |
|  | Model 1 | | | | Model 2 | | |
|  | Odds Ratio |  | 95% CI |  | Odds Ratio |  | 95% CI |
|  |  |  |  |  |  |  |  |
| Ever experienced child's death | 2.40 | * | [1.24, 4.67] |  |  |  |  |
|  |  |  |  |  |  |  |  |
| Timing of child's death |  |  |  |  |  |  |  |
| <=3 yrs |  |  |  |  | 3.51 | * | [1.10, 11.23] |
| 4-9 yrs ago |  |  |  |  | 2.98 | * | [1.10, 8.08] |
| 10+ years ago |  |  |  |  | 1.66 |  | [0.65, 4.28] |
| Never bereaved |  |  |  |  | -- |  | -- |
|  |  |  |  |  |  |  |  |
| Ever experienced child's near death | 1.71 | + | [0.97, 3.02] |  | 1.69 | + | [0.95, 2.99] |
|  |  |  |  |  |  |  |  |
| *Sociodemographic characteristics* |  |  |  |  |  |  |  |
| Ethnicity |  |  |  |  |  |  |  |
| Yao | -- |  | -- |  | -- |  | -- |
| Chewa | 0.88 |  | [0.27, 2.90] |  | 0.91 |  | [0.27, 3.00] |
| Lomwe | 0.76 |  | [0.30, 1.89] |  | 0.76 |  | [0.31, 1.91] |
| Tumbuka | 1.00 |  | -- |  | 1.00 |  | -- |
| Ngoni | 1.37 |  | [0.70, 2.69] |  | 1.41 |  | [0.72, 2.77] |
| Sena | 1.15 |  | [0.14, 9.58] |  | 1.11 |  | [0.13, 9.40] |
| Tonga | 7.77 |  | [0.62, 9.68] |  | 7.53 |  | [0.58, 97.67] |
| Other | 0.77 |  | [0.08, 6.98] |  | 0.76 |  | [0.08, 6.92] |
| Women's educational attainment |  |  |  |  |  |  |  |
| Primary | -- |  | -- |  | -- |  | -- |
| Secondary | 0.55 |  | [0.26, 1.15] |  | 0.54 |  | [0.26, 1.14] |
| Tertiary | 0.51 |  | [0.05, 4.92] |  | 0.51 |  | [0.05, 4.93] |
| Marital status |  |  |  |  |  |  |  |
| Currently married | -- |  | -- |  | -- |  | -- |
| Previously married | 1.12 |  | [0.55, 2.29] |  | 1.16 |  | [0.57, 2.36] |
| Never married | 1.46 |  | [0.48, 4.49] |  | 1.49 |  | [0.48, 4.56] |
| Experienced pregnancy loss | 1.21 |  | [0.57, 2.59] |  | 1.33 |  | [0.61, 2.87] |
| Total number of children | 1.04 |  | [0.77, 1.40] |  | 1.05 |  | [0.78, 1.41] |
| Household goods index | 1.09 |  | [0.93, 1.28] |  | 1.10 |  | [0.93, 1.29] |
| Employment status |  |  |  |  |  |  |  |
| Does not work | -- |  |  |  | -- |  |  |
| Piece work | 1.32 |  | [0.61, 2.86] |  | 1.31 |  | [0.60, 2.85] |
| Temporary employment | 0.75 |  | [0.30, 1.90] |  | 0.75 |  | [0.30, 1.90] |
| Steady employment | 0.79 |  | [0.41, 1.50] |  | 0.80 |  | [0.42, 1.53] |
| Experienced IPV in prior year | 1.16 |  | [0.53, 2.54] |  | 1.19 |  | [0.54, 2.60] |
| Perceived HIV status | 1.00 |  | [0.93, 1.08] |  | 1.00 |  | [0.93, 1.08] |
| Women's age | 0.98 |  | [0.90, 1.08] |  | 0.98 |  | [0.90, 1.06] |
| Fair/poor health | 2.93 | *** | [1.60, 5.36] |  | 2.98 | *** | [1.60, 5.45] |
| Orphan | 0.54 | * | [0.31, 0.96] |  | 0.54 | * | [0.31, 0.96] |
| Mother's education |  |  |  |  |  |  |  |
| None | -- |  | -- |  | -- |  | -- |
| Primary | 0.87 |  | [0.43, 1.79] |  | 0.87 |  | [0.42, 1.77] |
| Secondary | 0.96 |  | [0.30, 3.09] |  | 0.93 |  | [0.29, 3.00] |
| Tertiary | 3.65 |  | [0.33, 39.70] | | 3.46 |  | [0.32, 37.82] |
| Don’t know | 0.71 |  | [0.18, 2.89] |  | 0.69 |  | [0.17, 2.83] |
| *Shocks◊* |  |  |  |  |  |  |  |
| Health declined | 1.67 |  | [0.86, 3.24] |  | 1.68 |  | [0.86, 3.26] |
| Affected by witchcraft | 1.26 |  | [0.27, 5.80] |  | 1.28 |  | [0.27, 5.99] |
| Had malaria | 1.40 |  | [0.79, 2.51] |  | 1.42 |  | [0.79, 2.53] |
| Hospitalized | 2.47 | ** | [1.25, 4.89] |  | 2.42 | * | [1.22, 4.78] |
| Food shortage | 1.55 |  | [0.78, 3.24] |  | 1.59 |  | [0.76, 3.35] |
| Lost job/worse job | 1.08 |  | [0.32, 3.65] |  | 1.07 |  | [0.32, 3.56] |
| Partner infidelity | 1.12 |  | [0.61, 2.06] |  | 1.13 |  | [0.61, 2.08] |
| Family member illness | 1.47 |  | [0.71, 3.03] |  | 1.49 |  | [0.72, 3.08] |
| Number of funerals attended | 1.04 |  | [0.84, 1.28] |  | 1.03 |  | [0.83, 1.27] |
| Household bereavement | 1.40 |  | [0.77, 2.56] |  | 1.38 |  | [0.75, 2.54] |
|  |  |  |  |  |  |  |  |
| LR Test | 81.13 | *** |  |  | 82.50 | *** |  |
| NOTE: TLT-3 data; N=1,416 |  |  |  |  |  |  |  |
| ****p*<.001; ***p*<.01; **p*<.05; +*p*<.1 |  |  |  |  |  |  |  |
| *◊*Shocks are measured within the past four months except for funeral attendance, which pertains to the prior month. | | | | | | | |

| TABLE A2 Ordinal logistic regression model results of women’s frequency of ‘feeling depressed’ in the prior month among mothers in Balaka, Malawi, enrolled in the Tsogolo La Thanzi-3 study, expressed as odds ratios | | | | | | | |
| --- | --- | --- | --- | --- | --- | --- | --- |
| Variable | Model 1 | | |  | Model 2 | | |
|  | Odds Ratio [95% CI] | | |  | Odds Ratio [95% CI] | | |
|  |  |  |  |  |  |  |  |
| Ever experienced child's death | 1.29 |  | [0.93, 1.78] | |  |  |  |
|  |  |  |  |  |  |  |  |
| Timing of child's death |  |  |  |  |  |  |  |
| <=3 yrs |  |  |  |  | 2.32 | * | [1.19, 14.51] |
| 4-9 yrs ago |  |  |  |  | 0.90 |  | [0.52, 1.56] |
| 10+ years ago |  |  |  |  | 1.29 |  | [0.83, 1.99] |
| Never bereaved |  |  |  |  | -- |  | -- |
|  |  |  |  |  |  |  |  |
| Ever experienced child’s near death | 1.46 | ** | [1.16, 1.84] | | 1.47 | ** | [1.17, 1.86] |
|  |  |  |  |  |  |  |  |
| *Sociodemographic characteristics* |  |  |  |  |  |  |  |
| Ethnicity |  |  |  |  |  |  |  |
| Yao | -- |  | -- |  | -- |  | -- |
| Chewa | 1.25 |  | [0.78, 2.00] | | 1.27 |  | [0.79, 2.03] |
| Lomwe | 1.19 |  | [0.85, 1.66] | | 1.19 |  | [0.85, 1.66] |
| Tumbuka | 1.09 |  | [0.32, 3.77] | | 1.13 |  | [0.33, 3.91] |
| Ngoni | 1.15 |  | [0.87, 1.52] | | 1.17 |  | [0.88, 1.54] |
| Sena | 1.76 |  | [0.84, 3.68] | | 1.79 |  | [0.85, 3.75] |
| Tonga | 1.83 |  | [0.33, 10.24] | | 1.97 |  | [0.36, 10.74] |
| Other | 1.65 |  | [0.80, 3.42] | | 1.68 |  | [0.81, 3.48] |
| Women's educational attainment |  |  |  |  |  |  |  |
| Standard | -- |  | -- |  | -- |  | -- |
| Form | 1.48 | ** | [1.12, 1.95] | | 1.51 | ** | [1.14, 1.99] |
| Tertiary | 1.68 |  | [0.79, 3.57] | | 1.73 |  | [0.81, 3.70] |
| Marital status |  |  |  |  |  |  |  |
| Currently married | -- |  | -- |  | -- |  | -- |
| Previously married | 1.15 |  | [0.84, 1.57] | | 1.14 |  | [0.83, 1.56] |
| Never married | 1.4 |  | [0.89, 2.19] | | 1.39 |  | [0.89, 2.19] |
| Experienced pregnancy loss | 1.49 | * | [1.07, 2.08] | | 1.48 | * | [1.06, 2.06] |
| Total number of children | 0.88 | + | [0.78, 1.00] | | 0.88 | + | [0.78, 1.00] |
| Household goods index | 0.95 | + | [0.89, 1.01] | | 0.94 | + | [0.88, 1.01] |
| Employment status |  |  |  |  |  |  |  |
| Does not work | -- |  |  |  | -- |  |  |
| Piece work | 1.09 |  | [0.77, 1.56] | | 1.11 |  | [0.77, 1.58] |
| Temporary employment | 1.19 |  | [0.84, 1.68] | | 1.21 |  | [0.85, 1.71] |
| Steady employment | 0.83 |  | [0.64, 1.07] | | 0.82 |  | [0.63, 1.06] |
| Experienced IPV in prior year | 2.28 | *** | [1.61, 3.22] | | 2.30 | *** | [1.63, 3.25] |
| Perceived HIV status | 0.97 |  | [0.94, 1.01] | | 0.97 |  | [0.94, 1.01] |
| Women's age | 1.05 | * | [1.01, 1.08] | | 1.05 | * | [1.01, 1.08] |
| Fair/poor health | 2.38 | *** | [1.79, 3.16] | | 2.39 | *** | [1.80, 3.18] |
| Orphan | 1.02 |  | [0.81, 1.28] | | 1.03 |  | [0.82, 1.29] |
| Mother's education |  |  |  |  |  |  |  |
| None | -- |  | -- |  | -- |  | -- |
| Primary | 1.5 | ** | [1.11, 2.05] | | 1.50 | * | [1.10, 2.03] |
| Secondary | 1.5 |  | [0.92, 2.45] | | 1.49 |  | [0.91, 2.43] |
| Tertiary | 2.59 | + | [0.94, 7.12] | | 2.46 | + | [0.89, 6.78] |
| Don’t know | 1.17 |  | [0.66, 2.08] | | 1.18 |  | [0.66, 2.09] |
| *Shocks◊* |  |  |  |  |  |  |  |
| Health declined | 1.41 | * | [1.03, 1.94] | | 1.43 | * | [1.04, 1.96] |
| Affected by witchcraft | 1.99 | * | [1.07, 3.71] | | 2.00 | * | [1.08, 3.73] |
| Had malaria | 1.37 | * | [1.07, 1.76] | | 1.38 | * | [1.08, 1.78] |
| Hospitalized | 1.26 |  | [0.89, 1.80] | | 1.24 |  | [0.87, 1.77] |
| Food shortage | 1.71 | *** | [1.31, 2.22] | | 1.70 | *** | [1.31, 2.21] |
| Lost job/worse job | 1.78 | * | [1.06, 3.01] | | 1.73 | * | [1.03, 2.91] |
| Partner infidelity | 2.27 | *** | [1.74, 2.95] | | 2.30 | *** | [1.77, 3.00] |
| Family member illness | 1.09 |  | [0.85, 1.41] | | 1.10 |  | [0.85, 1.41] |
| Number of funerals attended | 0.96 |  | [0.88, 1.05] | | 0.96 |  | [0.88, 1.05] |
| Household bereavement | 1.67 |  | [1.27, 2.19] | | 1.63 |  | [1.24, 2.15] |
|  |  |  |  |  |  |  |  |
| LR test | 317.70 | | *** |  | 322.43 | | *** |
| NOTE: TLT-3 data; N=1,146 |  |  |  |  |  |  |  |
| ****p*<.001; ***p*<.01; **p*<.05; +*p*<.1 |  |  |  |  |  |  |  |
| *◊*Shocks are measured within the past four months except for funeral attendance, which pertains to the prior month. | | | | | | | |

| TABLE A3 Logistic regression model results of women’s odds of reporting ‘feeling depressed’ often in the prior month among mothers in Balaka, Malawi, enrolled in the Tsogolo la Thanzi-3 study, expressed as odds ratios | | | | | | | |
| --- | --- | --- | --- | --- | --- | --- | --- |
| Variable | Model 1 | | |  | Model 2 | | |
|  | Odds Ratio [95% CI] | | |  | Odds Ratio [95% CI] | | |
|  |  |  |  |  |  |  |  |
| Ever experienced child's death | 1.33 |  | [0.82, 2.16] | |  |  |  |
|  |  |  |  |  |  |  |  |
| Timing of child's death |  |  |  |  |  |  |  |
| <=3 yrs |  |  |  |  | 3.18 | * | [1.28, 7.88] |
| 4-9 yrs ago |  |  |  |  | 0.75 |  | [0.30, 1.85] |
| 10+ years ago |  |  |  |  | 1.28 |  | [0.68, 2.43] |
| Never bereaved |  |  |  |  | -- |  | -- |
|  |  |  |  |  |  |  |  |
| Ever experienced child’s near death | 1.73 | ** | [1.19, 2.53] | | 1.76 | ** | [1.20, 2.58] |
|  |  |  |  |  |  |  |  |
| *Sociodemographic characteristics* |  |  |  |  |  |  |  |
| Ethnicity |  |  |  |  |  |  |  |
| Yao | -- |  | -- |  | -- |  | -- |
| Chewa | 1.42 |  | [0.66, 3.07] | | 1.48 |  | [0.69, 3.20] |
| Lomwe | 1.28 |  | [0.70, 2.31] | | 1.27 |  | [0.70, 2.31] |
| Tumbuka | 5.21 | + | [0.88, 30.87] | | 5.46 | + | [0.90, 33.01] |
| Ngoni | 1.58 | + | [0.98, 2.53] | | 1.64 | * | [1.02, 2.64] |
| Sena | 2.03 |  | [0.53, 7.80] | | 1.96 |  | [0.49, 7.80] |
| Tonga | 1.53 |  | [0.11, 21.08] | | 1.88 |  | [0.14, 25.46] |
| Other | 1.54 |  | [0.41, 5.83] | | 1.62 |  | [0.43, 6.16] |
| Women's educational attainment |  |  |  |  |  |  |  |
| Standard | -- |  | -- |  | -- |  | -- |
| Form | 1.05 | ** | [0.66, 1.66] | | 1.08 | ** | [0.68, 1.71] |
| Tertiary | 0.32 |  | [0.04, 2.89] | | 0.33 |  | [0.04, 2.95] |
| Marital status |  |  |  |  |  |  |  |
| Currently married | -- |  | -- |  | -- |  | -- |
| Previously married | 1.67 | * | [1.05, 2.64] | | 1.65 |  | [1.04, 2.62] |
| Never married | 1.30 |  | [0.89, 2.19] | | 1.30 |  | [0.58, 2.92] |
| Experienced pregnancy loss | 1.66 | + | [0.58, 2.92] | | 1.66 | * | [0.99, 2.78] |
| Total number of children | 0.95 |  | [0.78, 1.16] | | 0.94 |  | [0.77, 1.15] |
| Household goods index | 0.94 |  | [0.84, 1.06] | | 0.95 |  | [0.85, 1.06] |
| Employment status |  |  |  |  |  |  |  |
| Does not work | -- |  |  |  | -- |  |  |
| Piece work | 1.23 |  | [0.71, 2.14] | | 1.26 |  | [0.72, 2.19] |
| Temporary employment | 1.39 |  | [0.79, 2.44] | | 1.42 |  | [0.81, 2.49] |
| Steady employment | 0.86 |  | [0.56, 1.35] | | 0.86 |  | [0.55, 1.34] |
| Experienced IPV in prior year | 2.65 | *** | [1.62, 4.32] | | 2.69 | *** | [1.65, 4.41] |
| Perceived risk HIV | 0.98 |  | [0.93, 1.03] | | 0.98 |  | [0.93, 1.03] |
| Women's age | 1.04 |  | [0.99, 1.10] | | 1.05 |  | [0.99, 1.10] |
| Fair/poor health | 2.48 | *** | [1.64, 3.74] | | 2.50 | *** | [1.65, 3.79] |
| Orphan | 0.98 |  | [0.67, 1.42] | | 1.00 |  | [0.69, 1.46] |
| Mother's education |  |  |  |  |  |  |  |
| None | -- |  | -- |  | -- |  | -- |
| Primary | 2.59 | ** | [1.43, 4.70] | | 2.58 | ** | [1.42, 4.70] |
| Secondary | 3.42 | ** | [1.46, 8.05] | | 3.41 | ** | [1.45, 8.04] |
| Tertiary | 5.03 | + | [0.83, 30.61] | | 4.66 | + | [0.77, 28.11] |
| Don’t know | 2.50 | + | [0.98, 6.38] | | 2.62 | * | [1.03, 6.69] |
| *Shocks◊* |  |  |  |  |  |  |  |
| Health declined | 0.96 |  | [0.59, 1.56] | | 0.97 | * | [0.59, 1.58] |
| Affected by witchcraft | 1.83 |  | [0.72, 4.68] | | 1.87 | * | [0.73, 4.80] |
| Had malaria | 1.28 |  | [0.86, 1.90] | | 1.29 | * | [0.87, 1.92] |
| Hospitalized | 1.05 |  | [0.60, 1.85] | | 1.02 |  | [0.58, 1.80] |
| Food shortage | 2.59 | *** | [1.56, 4.29] | | 2.58 | *** | [1.55, 4.28] |
| Lost job/worse job | 0.84 |  | [0.37, 1.93] | | 0.81 |  | [0.35, 1.87] |
| Partner infidelity | 2.47 | *** | [1.68, 3.64] | | 2.57 | *** | [1.74, 3.79] |
| Family member illness | 1.07 |  | [0.69, 1.66] | | 1.06 |  | [0.68, 1.66] |
| Number of funerals attended | 0.90 |  | [0.77, 1.04] | | 0.90 |  | [0.78, 1.05] |
| Household bereavement | 1.78 | ** | [1.18, 2.70] | | 1.71 | * | [1.12, 2.60] |
|  |  |  |  |  |  |  |  |
| LR test | 196.46 | | *** |  | 201.54 | | *** |
| NOTE: TLT-3 data; N=1,146 |  |  |  |  |  |  |  |
| ****p*<.001; ***p*<.01; **p*<.05; +*p*<.1 |  |  |  |  |  |  |  |
| *◊* Shocks are measured within the past four months except for funeral attendance, which pertains to the prior month. | | | | | | | |
